# Effects of Semaglutide and Tirzepatide on Bone Metabolism in Type 2 Diabetic Mice

**DOI:** 10.3390/ph17121655

**Published:** 2024-12-09

**Authors:** Fang Lv, Xiaoling Cai, Chu Lin, Wenjia Yang, Linong Ji

**Affiliations:** Department of Endocrinology and Metabolism, Peking University People’s Hospital, No.11 Xizhimen South Street, Xicheng District, Beijing 100044, China; lvfang1a@163.com (F.L.); chulin@bjmu.edu.cn (C.L.); ywjsunshine@126.com (W.Y.)

**Keywords:** GLP-1 receptor agonists, GIP/GLP-1 receptor agonists, diabetes, bone metabolism

## Abstract

**Background/Objectives:** Type 2 diabetes and weight loss are associated with detrimental skeletal health. Incretin-based therapies (GLP-1 receptor agonists, and dual GIP/GLP-1 receptor agonists) are used clinically to treat diabetes and obesity. The potential effects of semaglutide and tirzepatide on bone metabolism in type 2 diabetic mice remain uncertain. **Methods:** Combined streptozotocin and high fat feeding were employed in female C57BL/6J mice to promote hyperglycemia. Mice were administered for 4 weeks with a saline vehicle (sc., once-daily), semaglutide (40 μg/kg/d, sc., every three days), or tirzepatide (10 nmol/kg, sc., once-daily). Bone strength was assessed by three-point bending. Femur microarchitecture was determined by micro-CT, and bone formation and resorption parameters were measured by histomorphometric analysis. Serum was collected to measure bone resorption (C-telopeptide fragments of type I collagen, CTX) and formation (procollagen type 1 N-terminal propeptide, P1NP) biomarkers, respectively. The expression of bone metabolism-related genes was evaluated in the bone using RT-PCR. Results: Glucose levels significantly reduced after 4 weeks of semaglutide and tirzepatide treatment (both *p* < 0.05) compared with vehicle treatment. Tirzepatide led to more weight loss than semaglutide. Compared to saline-treated diabetic mice, the mean femur length was shorter in the tirzepatide group. After treatment with tirzepatide or semaglutide, cortical bone and trabecular bone parameters did not change significantly compared to saline-treated diabetic mice, except that cortical thickness was lower in the semaglutide group compared to the saline group (*p* = 0.032). Though CTX and P1NP levels decreased, however, the change in CTX and P1NP levels did not differ among the four groups during the 4 weeks of treatment (all *p* > 0.05). Semaglutide affected *RANKL* and *OPG* mRNA expression and increased the ratio of *OPG*/*RANKL*. No significant difference was found in the quantity of *Col1a1*, *RANKL*, *OPG*, and *RUNX2* between tirzepatide- and saline-treated diabetic mice. Conclusions: The 4-week treatment with semaglutide and tirzepatide had a neutral effect on bone mass compared with the controls, and most of the bone microarchitecture parameters were also comparable between groups in diabetic mice. A better understanding of incretin-based therapies on bone metabolism in patients with diabetes requires further evaluation in large clinical trials.

## 1. Introduction

Type 2 diabetes (T2D) has emerged as a leading public health concern worldwide. T2D increases the incidence of bone fracture in patients despite having increased bone mineral density (BMD) [1], and common fracture sites are the hip, feet, and proximal femur [2]. Impairments in bone material properties, increased cortical porosity, and low bone turnover have been observed in T2D [3]. Weight loss is a critical component of managing diabetes and is recommended for most patients with T2D who are classified as overweight or obese by the American Diabetes Association [4]. Although there are well-established beneficial effects of weight loss on improving glycemic control and reducing cardiovascular risk factors in these patients, weight loss has been associated with bone loss and potentially increased risk of fracture [5]. Therefore, the potential negative impact of weight loss on skeletal health has become a concern for the treatment of T2D, and thoughtful considerations for preserving bone are necessary for weight loss planning.

Anti-diabetic drugs have different effects on bone metabolism [6]. Of note, some antidiabetic drugs (e.g., thiazolidinediones, some SGLT2 inhibitors) may affect bone metabolism. Medications with a neutral or favorable effect on bone metabolism should be the preferred treatment in patients with T2D at increased fracture risk based on BMD testing and/or FRAX scores [7]. Glucagon-like peptide-1 receptor agonists (GLP-1 RAs) and dual hormones glucagon-like protein-1 (GLP-1) and glucose-dependent insulinotropic peptide (GIP) receptor agonists are amongst the most recent class of incretin-based drugs approved for the treatment of T2D and obesity [8], which is expected to exert potentially beneficial effects on bone health [9]. Interestingly, the latest meta-analysis of GLP-1 RAs, which included 38 studies with 39,795 patients, found that when compared with placebo and other antidiabetic drugs, liraglutide and lixisenatide were associated with a statistically significant reduction in the risk of fractures, and the beneficial effects were dependent on the duration of treatment [10]. To our knowledge, the therapeutic potential of semaglutide and tirzepatide for bone metabolism in type 2 diabetes remains uncertain.

In this study, we wanted to examine the skeletal effects of GLP-1 RA (semaglutide) and dual GLP-1/GIP RA (tirzepatide) in an animal model of T2D induced by joint administration of a high fat diet and streptozotocin (STZ). We investigated the effects of semaglutide and tirzepatide on bone mass, bone microarchitecture, bone turnover biomarkers, and the expression of bone metabolism-related genes.

## 2. Results

### 2.1. Semaglutide and Tirzepatide Treatment on Blood Glucose Level and Body Weight

**Figure 1:**
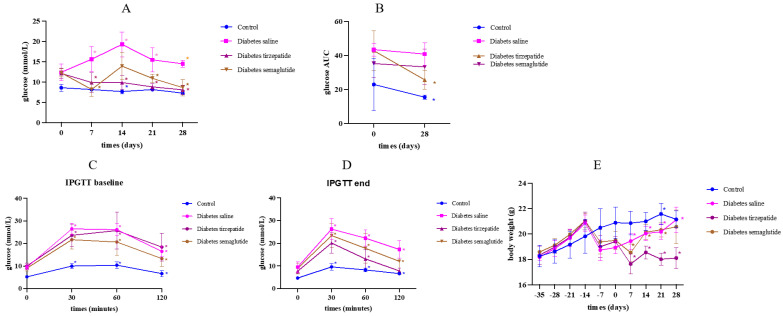
Blood glucose level and body weight. All data were presented as means ± SD. *: Compared to week 0, *p* < 0.05. (**A**) blood glucose fast of 5 h; (**B**) blood glucose levels during IPGTT at baseline; (**C**) blood glucose levels during IPGTT at the end of study; (**D**) area under the curve (AUC) for the blood glucose levels during IPGTT; (**E**) change in body weight.

Saline-treated diabetic mice presented with high blood glucose levels through the full course of this study. Glucose levels significantly reduced after 4 weeks of semaglutide and tirzepatide (both *p* < 0.05) (Figure 1A). IPGTT revealed that 4 weeks of treatment with tirzepatide significantly reduced glucose AUC (*p* = 0.007). However, treatment with saline and semaglutide did not reduce glucose AUC significantly (*p* = 0.203 and *p* = 0.445) (Figure 1B). The 4 weeks of treatment with semaglutide and tirzepatide significantly reduced glucose excursion during IPGTT (Figure 1C,D).

Body weight increased more in diabetic mice compared to non-diabetic mice in the first 3 weeks of the HFD, which might be due to a gain in fat mass. However, after 3 weeks of the HFD, body weight was still increased in non-diabetic mice, while body weight began to reduce in diabetic mice. After treatment with tirzepatide, body weight continued to drop. After treatment with semaglutide, body weight dropped in the first week, but then continued to gain (Figure 1E).

### 2.2. Semaglutide and Tirzepatide Treatment on Bone Turnover

**Figure 2:**
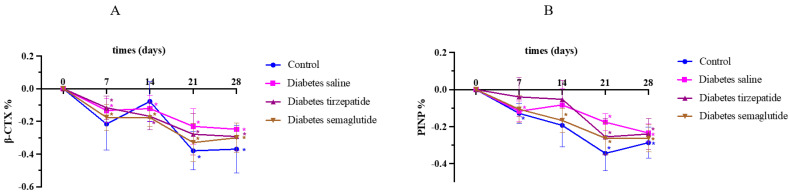
Change of bone turnover. All data are presented as means ± SD. *: Compared to week 0, *p* < 0.05. (**A**) levels of CTX; (**B**) levels of P1NP.

Compared with non-diabetic mice, diabetic mice exhibited higher levels of CTX and P1NP before treatment. At week 4, P1NP levels reduced in all diabetic mice and non-diabetic mice. The levels of P1NP in the semaglutide-treated group at week 1, and in the tirzepatide-treated group at week 1 and week 2 did not reduce significantly. However, the change in CTX and P1NP levels were similar to the change that was observed in saline-treated diabetic mice during 4 weeks of treatment (all *p* > 0.05) (Figure 2).

### 2.3. Semaglutide and Tirzepatide Treatment on Bone Microarchitecture

**Figure 3:**
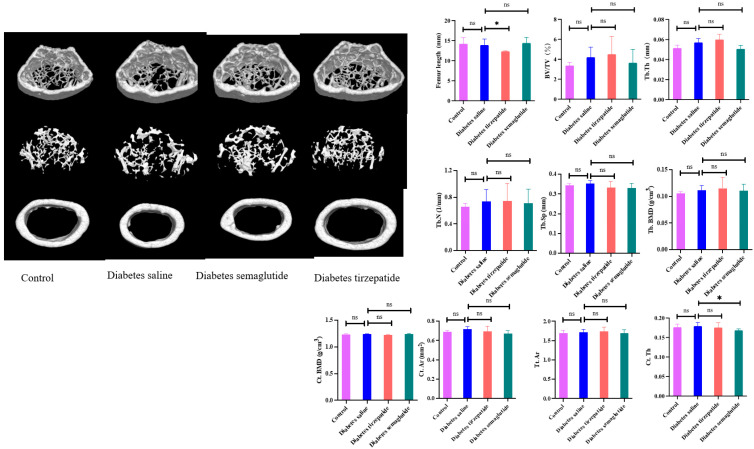
Micro-CT assessment of trabecular and cortical bone of femur from mice 4 weeks post-treatment. The constructed 3D images of trabecular bone and cortical bone of the femurs. The skeletal parameters included bone volume fraction (BV/TV), trabecular bone mineral density (Tb.BMD), trabecular thickness (Tb.Th), separation (Tb.Sp), and number (Tb.N), cortical BMD (Ct.BMD), cortical area (Ct.Ar), cortical thickness (Ct.Th), and total cross-sectional area (Tt.Ar). All data are presented as means ± SD. *: Compared to saline-treated diabetic mice, *p* < 0.05; ns, non-significant.

Compared to saline-treated diabetic mice, the mean femur length was shorter in the tirzepatide group (Figure 3). There were no significant differences in femur lengths between the saline group and the semaglutide group, or the non-diabetic group and the saline group. We examined the effects of semaglutide and tirzepatide on the cortical and trabecular bone microarchitectures of the femur. The BV/TV, Tb.BMD, Tb.Th, Tb.Sp, Tb.N, Ct.BMD, Ct.Ar, and Tt.Ar were similar in non-diabetic mice and saline-treated diabetic mice (all *p* > 0.05). After treatment with tirzepatide or semaglutide, cortical bone and trabecular bone parameters did not change significantly compared to saline-treated diabetic mice, except that Ct.Th was lower in the semaglutide group (*p* = 0.032).

### 2.4. Semaglutide and Tirzepatide Treatment on Bone Strength and Bone Histological Parameters

**Figure 4:**
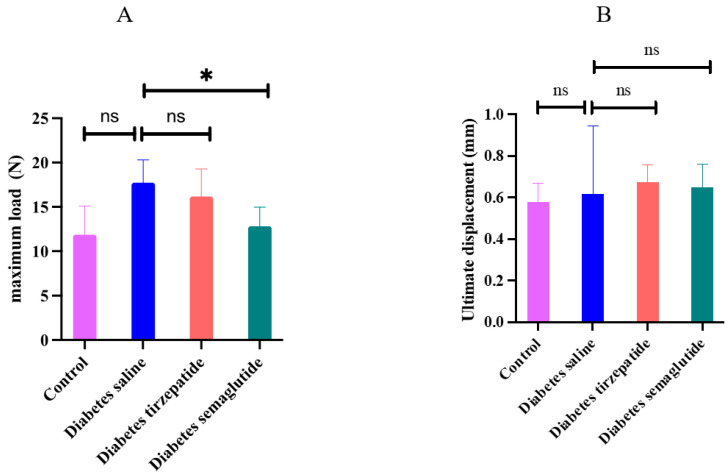
Maximum load and ultimate displacement determined by the load displacement curve. All data are presented as means ± SD. *: Compared to saline-treated diabetic mice, *p* < 0.05; ns, non-significant. (**A**) maximum load; (**B**) ultimate displacement.

We examined the effects of diabetes and pharmacological interventions with semaglutide and tirzepatide on bone strength at the femur midshaft (Figure 4). Semaglutide significantly reduced the maximum load as compared with saline-treated diabetic mice (*p* = 0.016). Both semaglutide and tirzepatide did not significantly change the ultimate displacement (*p* > 0.05).

**Figure 5:**
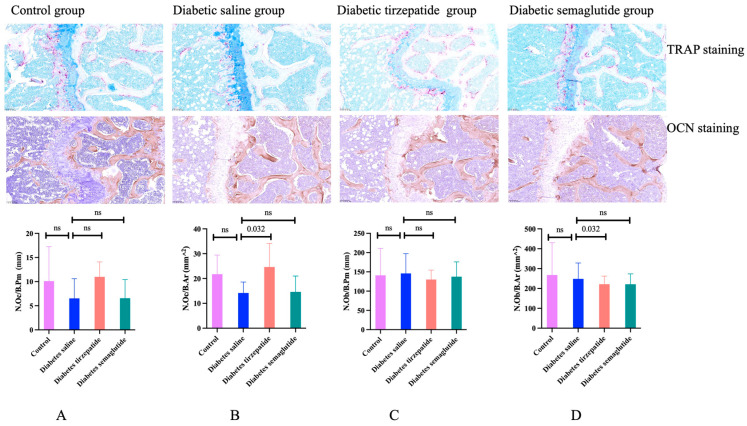
Osteocalcin (OCN) staining and tartrate-resistant acid phosphatase (TRAP) staining of femur. All data are presented as means ± SD. ns, non-significant. (**A**) osteoclast number per bone perimeter; (**B**) osteoclast number per bone area; (**C**) osteoblast number per bone perimeter; (**D**) osteoblast number per bone area.

TRAP staining and OCN staining were implemented to determine the osteoclast and osteoblast number per bone perimeter or per bone area. Compared to saline-treated diabetic mice, the tirzepatide treatment group showed a higher osteoclast number per bone area and lower osteoblast number per bone area. However, no other significance was found (Figure 5).

### 2.5. Semaglutide and Tirzepatide Treatment on Expression of Bone Metabolism-Related Genes

**Figure 6:**
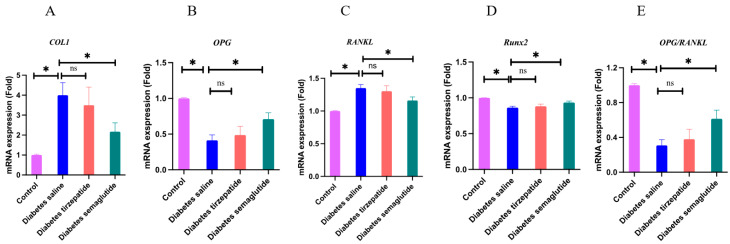
Results of real-time quantitative polymerase chain reaction. All data are presented as means ± SD. *: Compared to saline-treated diabetic mice, *p* < 0.05; ns, non-significant. (**A**) *Col1* mRNA; (**B**) *OPG* mRNA; (**C**) *RANKL* mRNA; (**D**) *RUNX2* mRNA; (**E**) *OPG/RANKL*.

Figure 6 shows the expression of *Col1*, *OPG*, *RANKL*, and *Runx2* mRNA after 4 weeks of treatment. Diabetic mice exhibited significantly higher *Col1* and *RANKL* mRNA expression and lower *OPG* and *RUNX2* mRNA expression than those without diabetes (*p* < 0.05). Compared with placebo treatment mice, mice treated with semaglutide exhibited significantly lower *Col1* and *RANKL* mRNA expression and higher *OPG* and *RUNX2* mRNA expression (*p* < 0.05). The results suggest that semaglutide increased the ratio of *OPG*/*RANKL*. No significant difference was found in the quantity of *Col1*, *RANKL*, *OPG*, and *RUNX2* between tirzepatide- and saline-treated diabetic mice.

## 3. Discussion

This study examined the effects of semaglutide and tirzepatide on bone metabolism in type 2 diabetic mice. We demonstrate that 4 weeks of treatment with semaglutide and tirzepatide had a neutral effect on bone mass compared with the controls, and most of the bone microarchitecture parameters were also comparable between groups in diabetic mice. We also showed that semaglutide affected *RANKL* and *OPG* mRNA expression and increased the ratio of *OPG*/*RANKL*, thereby inhibiting the process of osteoclastogenesis.

Type 2 diabetes can have detrimental effects on bone, and has been associated with an increased risk of fracture. The mechanisms underlying the impact of T2D on bone health are complex, some of which might have contradictory effects. For example, hyperglycemia in diabetes results in the accumulation of advanced glycation end products, which might disrupt bone turnover and deteriorate bone quality [11], whereas hyperinsulinemia in T2D increases osteoblast proliferation and bone formation [12]. Despite high or normal BMD, the bones of patients with diabetes has some structural characteristics, including greater cortical porosity, smaller cortical area, and decreased bone material strength, predisposing it to fractures [3,13]. However, there were no significant differences in bone mass or bone microarchitecture in this study. One possible reason is that short-term hyperglycemia might have little adverse effects on bone health. We also observed that diabetic mice exhibited significantly higher *Col1* (late markers of bone formation) and *RANKL* mRNA expression, and lower *OPG* and *RUNX2* (early markers of bone formation) mRNA expression than mice without diabetes. Therefore, persistent long-term hyperglycemia might affect bone resorption and bone formation, further reducing bone mass and bone strength, which needs to be verified in further studies.

Weight loss can reduce bone mass through decreased mechanical loading and decreased estrogen and other sex hormones, which can lead to a rise in bone resorption [14]. Changes in cytokines (such as interleukin-1, interleukin-6, and tumor necrosis factor-α), adipokines (such as leptin and adiponectin), and calcium and vitamin D deficiency may also play a role in bone metabolism changes during weight loss [15]. The mechanisms of bone health during weight loss in T2D might be more complicated than in those without T2D. Also, the contribution of each possible mechanism might vary by the different approaches used for weight loss [5,16]. The effects of incretin-based therapies (an attractive approach for the treatment of obesity) on bone health were inconsistent [17]. The GIP receptor is expressed in bone cells, and knockout of either GIP or its receptor induces severe bone quality alterations [18]. Similar alterations have also been observed in GLP-1 receptor knock-out animals associated with abnormal osteoclast resorption [19]. Double incretin receptor knockout (DIRKO) animals, lacking both the GIPR and GLP-1R, exhibited profound reductions in bone mass, and alterations of trabecular and cortical microarchitectures and tissue material properties [20]. Therefore, potential beneficial effects of both GIP and GLP-1 have been highlighted and might represent a suitable therapeutical option in the treatment of diabetes-induced bone fragility [17].

However, no incretin-based therapies for bone-related disorders are currently available, and the effects of existing incretin-based therapies on bone health have only recently started to emerge. Previous animal studies have explored the effects of GLP-1 RA on bone health in animal models with skeletal fragility. A 16-week administration of exenatide in ovariectomy (OVX)-induced osteoporosis in old rats was capable of enhancing bone strength and preventing the deterioration of trabecular microarchitecture by dual regulatory effects on bone turnover [21]. A 4-week treatment of liraglutide (0.3 mg/kg/day) and exenatide (10 μg/kg/day) in ovariectomised mice significantly increased trabecular bone mass, connectivity, and structural parameters, but had no effect on cortical bone [22]. However, administration of liraglutide (1.8 mg/kg/day) for 8 weeks in ovariectomized rats without diabetes significantly improved trabecular and cortical microarchitecture [23]. Clinically, incretin-based therapies in general have not been associated with safety issues regarding fractures. In recent years, the effects of GLP-1 RA on bone mass and bone turnover have been reported from relatively short and small randomized clinical trials in patients with T2D. Liraglutide treatment for 26 weeks in patients with T2D did not affect bone resorption and preserved hip BMD despite weight loss in patients with T2D [24]. In another study, patients with T2D were treated with exenatide and dulaglutide for 52 weeks. In the exenatide group, the BMD of the total hip increased. In the dulaglutide group, only the BMD of the femoral neck decreased, but the magnitude of decrease was less than that in the placebo group; the BMD of L1-L4, femoral neck, and total hip decreased significantly in the placebo group [25]. No studies have explored the effects of semaglutide on bone health. In this study, we found that the body weight did not change significantly with semaglutide treatment. Cortical bone and trabecular bone parameters and bone mechanical properties did not change significantly, except that Ct.Th and maximum load was lower in the semaglutide group compared to the saline group.

Recently, incretin-based double and triple agonists have been designed and successfully synthesized. As the use of double and triple agonists seems promising, it would be a prerequisite to ascertain their effects on bone health in more detail [17]. However, there are currently no studies assessing these drugs for bone health. Similar to recent clinical trials, in the present study we showed that tirzepatide, a dual agonist of receptors for GLP-1 and GIP, yielded more weight loss than semaglutide [7]. Although tirzepatide-treated diabetic mice had a shorter femur length compared to saline-treated diabetic mice, there were no significant differences in bone mass, bone microarchitecture, and bone strength between saline-treated and tirzepatide-treated diabetic mice.

After confirming the direct effect of semaglutide and tirzepatide on bone mass and bone strength, we assessed the effect of semaglutide and tirzepatide on biochemical markers and bone histology to further explore the possible underlying mechanisms of the possible anti-osteoporotic effects. In the present study, bone histomorphometric analyses showed that the number of osteoclasts and osteoblasts per bone perimeter were not significantly changed. Our results displayed that compared with saline-treated diabetic mice, semaglutide significantly increased *Runx2* mRNA levels, a key transcriptional factor that could stimulate the expression of other osteoblast-specific genes during the early stage of osteogenesis but not *Col1*, suggesting its different effects on the various stages of bone formation. The ratio of *OPG*/*RANKL* expression was believed to be a key determinant of osteoclastogenic activity. We found that semaglutide increased *OPG* mRNA expression and reduced *RANKL* mRNA expression, suggesting that semaglutide inhibited osteoclast differentiation by increasing the *OPG*/*RANKL* ratio. Tirzepatide did not change the gene expression level related to bone formation and bone resorption. These findings indicated that semaglutide might have bone-preserving effects in diabetic mice, which were possibly derived from its inhibiting effect on osteoclastic bone resorption. We observed a significant reduction in CTX levels following semaglutide and tirzepatide administration, but these reductions were similar to the change observed in saline group. In addition, despite a significant reduction in CTX levels with semaglutide and tirzepatide treatment, no effects of these therapies were observed in enhancing bone mechanical resistance and bone microstructure. Therefore, the effects of incretin-based therapies on bone metabolism in human remain to be addressed in the future.

There are some limitations in this study. Firstly, the experimental period of this study was only 4 weeks, which might not be able to adequately evaluate the anti-osteoporotic effects of semaglutide and tirzepatide. Insufficient increases in glucose and body weight were some other potential obstacles in evaluating the anti-osteoporotic effects of semaglutide and tirzepatide. In addition, body weight was reduced between −14 and −7 days for diabetic mice in this study, which might be related to STZ injection and hyperglycemia. Studies with a longer duration, and mice with higher glucose and greater body weight are needed to assess effects of these therapies on bone health. Secondly, we have observed that women pay more attention to weight gain and obesity, especially young women. However, weight loss in young people tends to cause lower peak bone mass, leading to a higher risk of osteoporosis after menopause. Therefore, we aimed to assess the effect of semaglutide and tirzepatide in female mice. Whether similar conclusions could be observed in male mice remain to be investigated. Further studies are needed with mice of different ages and genders. The effects of GLP-1, GIP, and dual/triple receptor agonists on fracture risk require further evaluation in large clinical trials as a primary outcome. Thirdly, different doses of semaglutide and tirzepatide would be different for weight loss and glycemic control. We did not set different dose groups so that we could not examine the effect of different doses of semaglutide and tirzepatide on bone metabolism. Fourthly, type 2 diabetes-associated lipid disorder is also associated with bone metabolism. However, we did not measure triglycerides and cholesterol in our study.

## 4. Materials and Methods

### 4.1. Animals

Eight-week-old female C57BL/6J mice (The Jackson Laboratory, Bar Harbor, ME, USA) were housed as 5 mice/cage. They received water ad libitum and were exposed to a 12 h light/dark cycle and 10% humidity at 20 ± 4 °C. The mice were adaptively fed for one week. Mice were fed a standard diet (normal control group, *n* = 8) or high-fat diet (HFD, 60 kcal% fat, D12492) (*n* = 30) throughout the experiment. After 2 weeks, mice in the HFD group were injected with STZ (50 mg/kg; Sigma-Aldrich, St Louis, MO, USA) i.p. for 8 consecutive days, while the mice in the normal control group were injected i.p. with citrate–phosphate buffer. Blood glucose levels were tested and mice with fasted blood glucose levels above 8.3 mmol/L (150 mg/dL) were considered to be type 2 diabetic mice.

**Figure 7:**
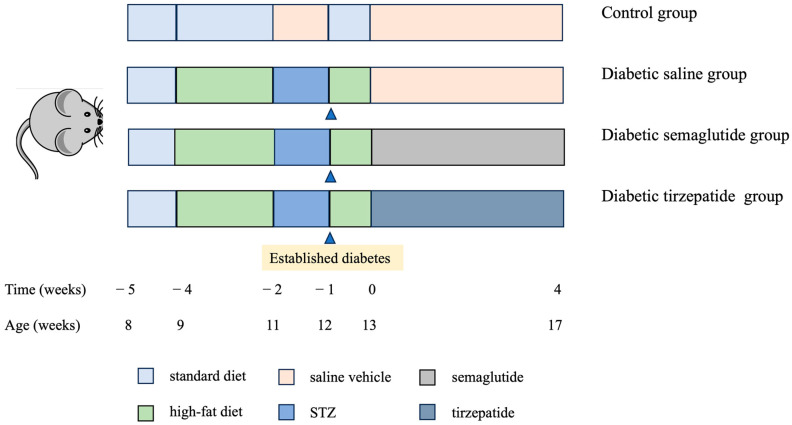
Schematic of the experimental design for animal study. At 13 weeks of age, mice were subcutaneously injected with either saline, semaglutide, or tirzepatide until 17 weeks of age (4 week treatment period).

The diabetic mice were then randomly separated into three groups (*n* = 10/group) and were subcutaneously injected with either saline, semaglutide, or tirzepatide for 4 weeks until week 17: group 1—diabetic mice with saline vehicle (0.9% NaCl, sc., once-daily); group 2—diabetic mice with semaglutide (40 μg/kg, subcutaneous, every three days); group 3—diabetic mice with tirzepatide (10 nmol/kg, subcutaneous, once-daily). Normal control mice were injected with the saline vehicle (Figure 7). These doses of semaglutide and tirzepatide were based on previous studies [26,27]. The period of 4 weeks was previously shown to induce significant improvement in tissue material properties [28].

Through the course of this study, body weight and blood glucose levels were assessed every week in mice fasted for 5 h. Food consumption was recorded weekly. At the start and the end of the study, mice were fasted overnight for intraperitoneal glucose tolerance testing (IPGTT). Blood glucose levels were measured at 0, 30, 60, and 120 min after 20% glucose (2 mg/g) intraperitoneal injection. The area under the curve (AUC) for blood glucose was calculated by the linear trapezoidal method for both IPGTT. At necropsy, femurs and tibia were cleaned of soft tissues and stored either in saline-soaked gauze at −20 °C (bone strength assessment) or in 70% ethanol at 4 °C (bone histology and histomorphometry) until used.

### 4.2. Biochemical Analyses

Blood was collected at baseline and every week from the facial vein of 5 h fasted mice. Procollagen type 1 N-terminal propeptide (P1NP) and C- telopeptide fragments of type I collagen (CTX) (MEIMIAN, Jiangsu Meimian industrial Co., Ltd., Jiangsu, China) were measured following the manufacturer’s instructions.

### 4.3. Micro-Computed Tomography (Micro-CT) Analysis

The right femur (*n* = 5) from each group were scanned at an isotropic voxel resolution of 6.5 μm, 60 kV x-ray tube voltage, 200 μA tube electric current, and 2000 ms exposure time using SkyScan 1276 (Bruker, Kontich, Belgium). All cross-sectional images were reconstructed using 0–0.11 greyscale (NRecon Software version 1.1.17; Bruker, Kontich, Belgium). Analysis of bone morphometric parameters was performed by CTAn Software version 1.17.7.2 (Bruker, Kontich, Belgium). The trabecular volume of interest (VOI) was located 0.5 mm above the distal growth plate and extended on 1 mm up to the diaphysis. Cortical bone was analyzed in a 1000 μm long volume below the VOI of the trabecular bone situated in the middle of the diaphysis. Major bone morphometric parameters calculated by CTAn were reported, including bone volume fraction (BV/TV), trabecular bone mineral density (Tb.BMD), trabecular thickness (Tb.Th), separation (Tb.Sp), and number (Tb.N), cortical BMD (Ct.BMD), cortical area (Ct.Ar), cortical thickness (Ct.Th), and total cross-sectional area (Tt.Ar). All micro-CT parameters were determined according to guidelines and nomenclature proposed by the American Society for Bone and Mineral Research [29].

### 4.4. Biomechanical Testing

Three-point bending experiments were performed on left femurs after rehydrating bones at 4 °C for 24 h and letting them warm up to room temperature (22 ± 2 °C) for at least 6 h. Femur length was measured with digital caliper using anatomical reference point (great trochanter, femoral condyle). Three measurements were taken for each sample to calculate the average. Femurs were loaded to failure at room temperature at 2 mm/min using Electronic Universal Testing Machine WD-1 (Changchun Research Institute for Testing Machines, Changchun, Jilin, China). The lower span length was set to 10.5 mm. All force and displacement data were recorded until the specimen was broken. Maximum load (N; the load at the maximum failure point) and ultimate displacement (mm) were determined by the load displacement curve.

### 4.5. Bone Histomorphometric Analysis

Right femur samples were fixed with 4% paraformaldehyde (PFA), embedded in paraffin after decalcification, and made into 5-μm- thick sections. Hematoxylin and eosin (H&E) staining was used to show the histological performance. Tartrate-resistant acid phosphatase (TRAP) was performed to assess osteoclast activity status. The osteoclast number per bone perimeter (N.Oc/B.Pm) and osteoclast number per bone area (N.Oc/B.Ar) were calculated using Qupath v0.4.3 software (Queen’s University Belfast, UK). Immunostaining for osteocalcin (Ocn) was performed to determine the number of osteoblasts. Osteoblast number/bone perimeter (N.Ob/B.Pm) and osteoblast number per bone area (N.Ob/B.Ar) were quantified using Qupath software.

### 4.6. Quantitative Real-Time PCR (RT-PCR)

The total RNA content of the bone was measured by extraction from the tibia using TRIzol reagent (Invitrogen, Carlsbad, CA, USA) and was reverse transcribed to cDNA utilizing the GoScript Reverse Transcription Mix, Oligo(dT) Kit (Promega). The qPCR was performed using SYBR Green mix kit (Qiagen, Valencia, CA, USA). Specific primers for the *Runx2*, collagen type 1 (*Col1*), osteoprotegerin (*OPG*), receptor activator of NF-kB ligand (*RANKL*), and glyceraldehyde-3-phosphate dehydrogenase (*GAPDH*) (a housekeeping gene) were shown in Table 1. Relative mRNA expression levels were calculated using the 2^−ΔΔCT^ method (cycle threshold method) and normalized to the internal control *GAPDH*.

### 4.7. Statistical Analysis

Results were reported as means ± SD (standard difference). Comparisons of parameters between each group were completed using Student’s *t* test. Parameters of mice in different treatment groups were compared with One-way ANOVA and Tukey’s multiple comparison tests. Statistical analysis was conducted with SPSS version 25.0 software (SPSS Inc., Chicago, IL, USA). Statistical significance was determined when *p* values were equal to or less than 0.05.

## 5. Conclusions

Tirzepatide led to more weight loss than semaglutide. Though tirzepatide increased osteoclast number per bone area and reduced osteoblast number per bone area, semaglutide increased the ratio of *OPG*/*RANKL* compared to saline-treated diabetic mice. Semaglutide and tirzepatide had a neutral effect on bone mass compared with the controls, and most of bone microarchitecture parameters were also comparable between groups in diabetic mice. More studies are still required to validate these findings and to examine the underlying mechanisms. A better understanding of the actions of these gut hormones on bone homeostasis in patients with diabetes might lead to new strategies for the prevention and treatment of skeletal frailty related to diabetes.

## Figures and Tables

**Table 1:** Primers used for quantitative real-time PCR.

Gene		Primer Sequence (50–30)	Product Length (bp)
*COL1*	F	TCAGCTGCATACACAATGGC	117
	R	CATTGCATTGCACGTCATCG	
*OPG*	F	TGGTGCTCCTGGACATCATT	142
	R	CTCACTGTGCAGTGCTGTTT	
*RANKL*	F	AGCGCAGATGGATCCTAACA	130
	R	GCAGGAGTCAGGTAGTGTGT	
*RUNX2*	F	CTCTGGCCTTCCTCTCTCAG	150
	R	GTAGGTAAAGGTGGCTGGGT	
*GAPDH*	F	GGTGAAGGTCGGTGTGAACG	233
	R	CTCGCTCCTGGAAGATGGTG	

*COL1*: collagen type I alpha 1 chain; *OPG*: osteoprotegerin; *RANKL*: receptor activator of nuclear factor-κB ligand; *RUNX2*: runt-related transcription factor 2; *GAPDH* = glyceraldehyde-3-phosphate dehydrogenase.

## Data Availability

All data obtained throughout this study are incorporated within the manuscript.
